# Kundenseitige Akzeptanz digitaler Finanzassistenten: Empirische Untersuchung zur Ableitung von Handlungsempfehlungen für die Versicherungspraxis

**DOI:** 10.1007/s12297-022-00533-4

**Published:** 2022-10-10

**Authors:** Sascha Kwasniok, Stefan Bora

**Affiliations:** 1 Studienrichtung BWL-Versicherung, Duale Hochschule Baden-Württemberg (DHBW) Mannheim, Käfertaler Str. 258, 68167 Mannheim, Deutschland; 2 ALH Gruppe, Alte-Leipziger-Platz 1, 61440 Oberursel, Deutschland

**Keywords:** Financial Home, Technologieakzeptanz, PSD2, Regressionsanalyse, Faktorenanalyse, Financial home, Technology acceptance, PSD2, Regression analysis, Factor analysis

## Abstract

Die vorliegende Studie untersucht, welche Faktoren auf Kundenseite die Nutzungseinstellung zu digitalen Finanzassistenten beeinflussen. Digitale Finanzassistenten bilden dabei Online-Anwendungen, die Nutzern über mehrere Banken und Versicherer eine integrierte Darstellung ihrer gesamten Finanz- und Absicherungssituation erlauben. Für die Untersuchung werden Erkenntnisse der Technologieakzeptanzforschung (vor allem „Technology Acceptance Model (TAM)“) und der Forschung zur Nutzung internetbasierter Technologien (vor allem „Internet Users’ Information Privacy Concern (IUIPC)“) verwendet. Das abgeleitete Erklärungsmodell wird auf Grundlage einer Online-Befragung (*n* = 2054) zunächst überprüft. Anschließend erfolgt eine explorative Modellmodifikation mittels einer Faktorenanalyse über alle verwendeten Frage-Items. Die Ergebnisse der durchgeführten multiplen linearen Regressionsanalysen zeigen, dass der wahrgenommene Nutzen, das Anbietervertrauen sowie die persönliche Innovationsbereitschaft und die wahrgenommene Kompatibilität mit dem eigenen Lebensstil einen signifikanten Einfluss auf die Einstellung zu digitalen Finanzassistenten nehmen. Die Studienergebnisse bilden die Grundlage für die Ableitung von Handlungsempfehlungen für die Versicherungspraxis bezüglich der Gestaltung und Ausrichtung digitaler Finanzassistenten.

## Abstract (en)

This study analyses factors that influence customer’s attitude of using digital financial assistants. Digital financial assistants are online applications that allow users an integrated view of their entire financial and insurance situation over several banks and insurers. The study uses findings from technology acceptance research (especially “Technology Acceptance Model (TAM)”) as well as research on the use of internet-based technologies (especially “Internet Users’ Information Privacy Concern (IUIPC)”). The developed model is empirically tested by an online panel (*n* = 2054), followed by an exploratory modification by means of a factor analysis addressing all question items used. The results of the multiple linear regression analysis show that perceived usefulness, trusting beliefs as well as personal innovativeness and perceived compatibility with user’s own lifestyle have a significant influence on attitudes of using digital financial assistants. Based on this findings implications for insurance companies with regard to developing digital financial assistants are discussed.

## Einordnung digitaler Finanzassistenten in Theorie und Praxis

Mit der Umsetzung der zweiten Stufe der Payment Service Directive 2 (PSD2) im Jahr 2019 wurde der regulatorische Rahmen für eine Öffnung kundenbezogener Bankdaten geschaffen. So wurde vor allem mit sogenannten Kontoinformationsdienstleistern eine Rolle etabliert, die es Drittanbietern bei Vorliegen eines entsprechenden Kundenauftrags (sogenannter Consent) erlaubt, automatisiert und gebührenfrei auf die Kontoinformationen (z. B. Kontostände, Transaktionsdaten) einer oder mehrerer kontoführenden Banken zuzugreifen. Der regulatorisch vorangetriebenen Öffnung von Bankdaten (sogenanntes „Open Banking“; z. B. Guibaud 2016; Zachariadis und Ozcan 2017; Dratva 2020) liegt die Absicht zugrunde, die Entwicklung innovativer Finanzdienstleistungsangebote zu fördern und damit den Wettbewerb auf dem Finanzdienstleistungsmarkt zu stärken (Europäisches Parlament 2015, Art. 33).^1^

**Figure 1:**
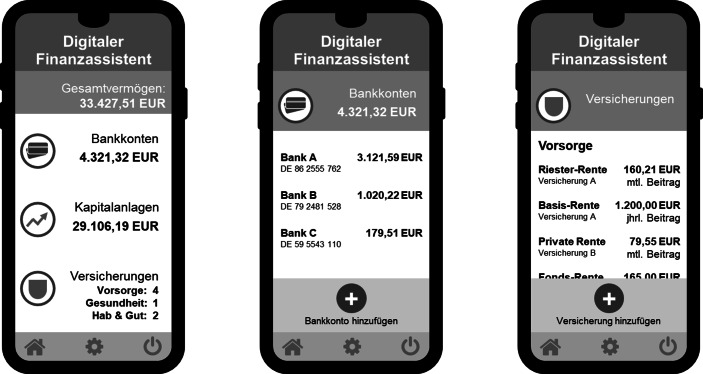

Als Ausprägung eines solchen innovativen Angebots ist in letzter Zeit die Entstehung digitaler Finanzassistenten zu beobachten. Digitale Finanzassistenten bilden onlinenutzbare Anwendungen, die Kunden einen umfänglichen Überblick über ihre persönliche Finanzsituation liefern (Wojahn und Kottmann 2020). Dazu werden Informationen beispielweise zu existierenden Bankkonten (z. B. Zahlungsströme), Versicherungsverträgen (z. B. Tarife) oder sonstigen Kapitalanlagen (z. B. Wertpapierdepots) per Datenabruf oder manueller Eingabe in einer Web-Oberfläche zusammengeführt und so dem Kunden in Gänze dargestellt (Abb. 1). Neben einer übersichtlichen und gesamthaften Darstellung der Finanz- und Absicherungssituation in einer Anwendung können digitale Finanzassistenten auf Basis der bereitgestellten persönlichen Finanzinformationen ihre Nutzer dabei unterstützen, finanzielle Entscheidungen in Abhängigkeit der individuellen Lebenssituation zu verbessern (z. B. Identifizierung von Deckungslücken, Kategorisierung von Ausgaben) (Guibaud 2016). In der Praxis werden solche Anwendungen auch als „Financial Home“ bezeichnet (z. B. Gensch und Tahedl 2016; Wojahn und Kottmann 2020).

In jüngerer Zeit sind Entwicklungen beobachtbar, bei denen Versicherer die Vorteile eines „Open Bankings“ durch die Integration von Kontoinformationsdienstleistern in ihre Kundenportale verwerten.^2^ Neben ihren Versicherungsverträgen erhalten Kunden über ihren Versicherer so zusätzlich einen Überblick über bestehende Bankverbindungen bei anderen Kreditinstituten. Auf diesem Weg entwickeln sich ursprüngliche rein versicherungsbezogene Kundenportale zu gesamtheitlichen, digitalen Finanzassistenten, die Kunden über die Absicherung ihre Risikosituation hinaus mit weiteren Finanzinformationen und -dienstleistungen versorgen.

In Bezug auf die strategische Ausrichtung eines Versicherers lassen sich solche Entwicklungen vor allem mit zwei Überlegungen erklären. Einerseits schränkt die vergleichsweise einfache Kopierbarkeit der Kernleistung „Versicherungsschutz“ in Verbindung mit fehlenden Schutzrechten die Möglichkeiten einer wettbewerbsstrategischen Differenzierung gegenüber der Konkurrenz alleine auf Grundlage neuer oder innovativer Versicherungsprodukte ein (z. B. Müller 1995). Untersuchungen zu Geschäftsmodell-Entwicklungen deuten darauf hin, dass bei imitierbaren Kernprodukten das Angebot zusätzlicher digitaler Serviceleistungen einen Beitrag zum Aufbau zumindest temporärer Innovationsvorsprünge liefern kann (z. B. Sorescu 2017). Digitale Finanzassistenten sind als Teil eines solchen Serviceangebots eines Versicherers zu verstehen. Daneben eröffnen ergänzende Serviceleistungen die Möglichkeit regelmäßiger Kundenkontaktpunkte, die zu einer Stärkung der Kundenbeziehung außerhalb der eigentlichen Nutzung des Kernprodukts „Versicherungsschutz“ beitragen können (Salomann 2008).

Andererseits kann das Angebot digitaler Finanzassistenten als Einstieg in die Entwicklung datengetriebener Vertriebs- und Geschäftsmodelle angesehen werden, bei denen durch Analyse und Verbindung interner und externer Daten neue Produkt- oder Serviceangebote geschaffen werden (z. B. Hartmann et al. 2016; Fritsch und Krotova 2020). Beispielweise können Versicherer bei Vorliegen einer entsprechenden Nutzereinwilligung die Kontoinformationen dazu verwenden, möglicherweise bestehende Deckungslücken des Kunden zu identifizieren und mit einem passenden Produktangebot zu schließen (z. B. Premchand und Choudhry 2018; Kwasniok et al. 2021).

Die dargestellten Vorteile digitaler Finanzassistenten in Bezug auf Wettbewerbsdifferenzierung sowie Vertriebs- und Geschäftsmodell-Gestaltung lassen sich aus Sicht von Versicherungsunternehmen allerdings nur dann realisieren, wenn ihre Kunden auch dazu bereit sind, die angebotenen digitalen Anwendungen in Anspruch zu nehmen. Zur Akzeptanz neuer Technologien liegt ein breites Spektrum an wissenschaftlichen Untersuchungen vor, die sich mit verschiedenen Anwendungsfelder beschäftigen (z. B. Schepers und Wetzels 2007; Marangunić und Granić 2015). In Bezug auf den Finanzdienstleistungssektor lässt sich insbesondere eine intensive Auseinandersetzung mit einer kundenseitigen Technologieakzeptanz von Online-Banking (z. B. Lee 2009; Hossain et al. 2020), Mobile-Banking (z. B. Baptista und Oliveira 2015; Sharma 2019) und digitalen Zahlungsdienstleistern (Ginner 2018) feststellen. Untersuchungen in der Versicherungswirtschaft widmen sich zumeist einzelnen Fragestellungen wie beispielsweise der Kundenakzeptanz von Online-Versicherungsvertrieb (z. B. Bauer et al. 2002; McKechnie et al. 2006), Online-Services wie Self-Services in Kundenportalen (z. B. Juric et al. 2015) oder Chatbots (z. B. Rodríguez Cardona et al. 2021) sowie sogenannte Wearables (z. B. Fitnessarmbänder) im Rahmen verhaltensbasierter Tarife (z. B. Wiegard et al. 2019).

Eine empirische Analyse der Einflussfaktoren einer kundenseitigen Akzeptanz digitaler Finanzassistenten liegt nach Wissen der Autoren zurzeit nicht vor.^3^ Ausgehend von dieser Forschungslücke behandelt die vorliegende Untersuchung folgende Fragestellung:

*Welche Faktoren beeinflussen auf Kundenseite die Einstellung zur Nutzung digitaler Finanzassistenten?*

Zur Beantwortung dieser Forschungsfrage wird zunächst ein Modell entwickelt, das auf Erkenntnissen der Technologieakzeptanzforschung und auf Untersuchungen zu Privatsphärenbedenken bei der Nutzung internetbasierter Technologien basiert. Das entwickelte Modell bildet die Grundlage der Hypothesengenerierung (Kap. 2). Nach einer Vorstellung des Untersuchungsdesigns werden die Untersuchungsergebnisse diskutiert (Kap. 3). Die Ableitung von Handlungsempfehlungen für die Unternehmenspraxis sowie Anknüpfungspunkte für weitere Forschung schließen diese Studie (Kap. 4).

## Modell zur Untersuchung der Nutzungseinstellung zu digitalen Finanzassistenten

### Modelltheoretische Grundlagen

Digitale Finanzassistenten bieten auch aufgrund der regulatorischen Rahmenbedingungen der PSD2 eine vergleichsweise neue technische Lösung für eine integrierte, gesamthafte Darstellung der individuellen Finanzsituation eines Anwenders. Für die Untersuchung der Nutzungsabsicht solcher Finanzassistenten orientiert sich die vorliegende Studie am „Technology Acceptance Model (TAM)“. Das von *Davis et al.* 1989 entwickelte TAM diente ursprünglich dazu, die Akzeptanz neuer Informationstechnologien durch Mitglieder von Organisationen zu erklären. In den letzten Jahren unterlag das TAM nicht nur mehreren Modifizierungen und Weiterentwicklungen. So wurde der Einfluss sozialer Prozesse berücksichtigt (TAM2; Venkatesh 2000), später fand eine Ausdifferenzierung der Einflussfaktoren der wahrgenommenen Benutzerfreundlichkeit statt (TAM3; Venkatesh und Bala 2008). Das TAM wurde mittlerweile auch auf verschiedene Anwendungsbereiche einer Technologienutzung außerhalb organisationaler Kontexte angewendet (z. B. Kornmeier 2009; Juric et al. 2015; Ahmad 2018). Das TAM ist damit auch für Untersuchungen der Technologieakzeptanz privater Nutzer und Verbraucher geeignet (Pavlou 2003).

Die Aggregation der individuellen Finanzsituation in einer Financial-Home-Anwendung erfolgt i. d. R. über die Eingabe von Authentifizierungsdaten, die den internetbasierten Abruf von Finanzdaten auslösen. Teilweise können fehlende Einträge zu Versicherungen oder sonstigen Finanzanlagen auch manuell ergänzt werden. Eine solche Übermittlung persönlicher, mitunter sensibler Daten lenkt die Frage auf die Informationssicherheit und den Datenschutz bei der Nutzung digitaler Finanzassistenten. Für die Betrachtung dieser Aspekte orientiert sich die vorliegende Untersuchung am „Internet Users’ Information Privacy Concern (IUIPC)“-Modell, das von *Malhotra et al. *(2004) auf Grundlage des „Global Information Privacy Concerns (GIPC)“-Ansatz von* Smith et al. *(1996) entwickelt wurde.

Aus beiden Ansätzen werden Einstellungskonstrukte als unabhängige Variablen herangezogen, um die kundenbezogene Nutzungseinstellung zu digitalen Finanzassistenten als abhängige Variable zu erklären („attitude towards technology“, ATT). Die Nutzungseinstellung gibt die Akzeptanz oder Ablehnung einer Technologie an. Vor allem die Neuartigkeit digitaler Finanzassistenten, die eine aktuelle geringe Kundendurchdringung zur Folge hat, lässt es sinnvoll erscheinen, anstelle der tatsächlichen Nutzung auf die generelle Nutzungseinstellung abzustellen. Mit ihrer Messung lassen sich insofern Rückschlüsse auf die tatsächliche Verwendung einer Technologie ziehen, als die Einstellung zu einem bestimmten Verhalten stark positiv mit den Verhaltensabsichten und dem realisierten Verhalten korreliert ist (Kim und Hunter 1993). Entsprechend diesen Erkenntnissen begünstigt eine positive Einstellung zu digitalen Finanzassistenten auch ihre tatsächliche Nutzung.

Folgende latenten Einstellungskonstrukte finden für die Untersuchung der kundenseitigen Nutzungsakzeptanz digitaler Finanzassistenten als unabhängige Variablen Berücksichtigung:

### Wahrgenommener Nutzen und wahrgenommene Benutzerfreundlichkeit

Der wahrgenommene Nutzen („perceived usefullness“, PU) und die wahrgenommene Benutzerfreundlichkeit („perceived ease of use“, PEU) stellen im TAM die zentralen Faktoren dar, die auf die Einstellung zur Technologienutzung wirken, die ihrerseits die Nutzungsabsicht und das tatsächliche Verhalten beeinflusst (Davis et al. 1989).

Der wahrgenommene Nutzen beschreibt, welche Mehrwerte der Nutzer aus der Verwendung einer neuen Technologie erwartet. Für digitale Finanzassistenten kann der Nutzen beispielweise in einer vollständigen Übersicht über die eigene Finanzsituation liegen, die eine Verbesserung finanz- und absicherungsbezogener Entscheidungen unterstützt. Den Erkenntnissen der die Motivation erklärenden Selbstbestimmungstheorie von *Deci und Ryan *(2000) folgend kann der wahrgenommene Nutzen als externer Anreiz betrachtet werden. Die Ausprägung des Anreizes beeinflusst das Einstellungskonstrukt des Anwenders, inwieweit ein Verhalten in Gang gesetzt wird, die mit der Technologienutzung verbundenen Vorteile auch tatsächlich zu realisieren. Aus diesem unterstellten Zusammenhang zwischen wahrgenommenem Nutzen und Technologieeinstellung wird folgende Hypothese aufgestellt:

*H1a: Je höher der wahrgenommene Nutzen ist, desto positiver ist die Einstellung zur Nutzung digitaler Finanzassistenten*.

Die wahrgenommene Benutzerfreundlichkeit gibt die Einschätzung des Anwenders an, welche physischen, kognitiven oder sonstigen Anstrengungen bei der Verwendung einer Technologie anfallen (Davis et al. 1989). Hierunter fallen etwa Aspekte, welche Vorkenntnisse ein Nutzer für die Verwendung eines digitalen Finanzassistenten benötigt oder wie einfach sich individuelle Anpassungen innerhalb der Anwendung vornehmen lassen. Je höher die Benutzerfreundlichkeit in der Wahrnehmung des Nutzers gestaltet ist, desto geringer sind seine Anstrengungen bei der Verwendung der Financial-Home-Anwendung. Geringe wahrgenommene Anstrengungen tragen dazu bei, die erwarteten Kosten der Technologienutzung zu reduzieren. Folgt man den Erkenntnissen der verhaltenswissenschaftlichen Marketingforschung, nach denen Einstellungen und daraus abgeleitete Verhaltensweisen vielfach das Ergebnis von Kosten-Nutzen-Abwägungen bilden (z. B. Andreasen 2002), kann hieraus ein positiver Einfluss der wahrgenommenen Benutzerfreundlichkeit auf die Einstellung zur Technologienutzung abgeleitet werden. So sollte mit steigender Benutzerfreundlichkeit der Technologie die Kosten-Nutzen-Bewertung der Technologienutzung günstiger ausfallen. Aus diesen Überlegungen ergibt sich nachstehende Hypothese:

*H1b: Je positiver die Benutzerfreundlichkeit wahrgenommen wird, desto positiver ist die Einstellung zur Nutzung von digitalen Finanzassistenten.*

### Wahrgenommene Kompatibilität und persönliche Innovationsbereitschaft

Mit der wahrgenommenen Kompatibilität („perceived compability“, PC) und der persönlichen Innovationsbereitschaft („personal innovativeness“, PI) werden im Rahmen dieser Untersuchung zwei zusätzliche Einflussfaktoren berücksichtigt, die die Technologieeinstellung mit Aspekten erklärt, die in der Person des Nutzers liegen. Beide Faktoren sind nicht Bestandteile des ursprünglichen TAM, finden aber in verschiedenen Erweiterungen des Grundmodells Eingang (z. B. Agarwal und Prasad 1998; Cho 2006; Schierz et al. 2010).

Wahrgenommene Kompatibilität beschreibt den Grad, bis zu dem die Technologienutzung als vereinbar mit bisher existierenden Erfahrungen, Werten und Bedürfnissen angesehen wird (Rogers 2003; Schierz et al. 2010). Die Bewertung der Technologie erfolgt damit nicht auf Basis technisch-rationaler Aspekte, sondern hinsichtlich des Effekts auf individuelle geprägte Wertekategorien der Nutzer (Thim 2017). Insofern liefert dieses Einstellungskonstrukt Rückschlüsse auf die persönliche Lebenseinstellung und den individuellen Lebensstil. Es ist plausibel anzunehmen, dass eine zunehmende Deckungsgleichheit der Funktionen digitaler Finanzassistenten mit der persönlichen Lebenseinstellung und dem individuellen Lebensstil die Einstellung zu dieser Technologie begünstigt. Dieser unterstellte Zusammenhang führt zu folgender Hypothese:

*H2a: Je höher die wahrgenommene Kompatibilität ist, desto positiver ist die Einstellung zur Nutzung von digitalen Finanzassistenten.*

Ein weiterer Aspekt, der in der Person des Anwenders liegt, ist dessen Innovationsbereitschaft. Bezogen auf die Technologienakzeptanz beschreibt dieser Einflussfaktor die Einstellung gegenüber neuen Technologien und den Grad der Bereitschaft, diese auch tatsächlich zu nutzen (Agarwal und Prasad 1998). Untersuchungen zur Innovationsdiffusion zeigen, dass eine hohe wahrgenommene Innovationsbereitschaft einen positiven Einfluss auf die Einstellung zur Technologienutzung ausübt (Rogers 2003). Erklären lässt sich dieser Zusammenhang mit dem ausgeprägten Interesse, technische Neuerungen vor anderen zu nutzen. Geleitet wird ein solches Verhalten von der Motivation, sich von der breiten Masse der Nutzer abzugrenzen, indem die Aufgeschlossenheit für Neues und das technologische Verständnis signalisiert wird (Dattée und Weil 2007; Fazel 2014). Die entsprechende Untersuchungshypothese wird folgendermaßen formuliert:

*H2b: Je höher die persönliche Innovationsbereitschaft ist, desto positiver ist die Einstellung zur Nutzung digitaler Finanzassistenten*.

### Individuelle Vertrauens- und Risikoüberzeugung

Die Nutzung von Financial-Home-Anwendungen setzt voraus, dass persönliche Daten (z. B. Zugangsdaten, Bank- oder Versicherungsdaten) durch Nutzung von Internettechnologien übermittelt werden. Mit einer Datenübermittlung im Internet eng verbunden ist das Bedürfnis nach einem Schutz der Privatsphäre (Viseu et al. 2004). In einem grundlegenden Verständnis beschreibt Privatsphäre die Möglichkeit, selbstbestimmt zu entscheiden, wann, wie und in welchem Umfang persönliche Informationen erhoben, weitergeben und weiterverarbeitet werden (Westin 1967). Die Untersuchung der Privatsphäre hinsichtlich der Einstellung zur Verwendung von Financial-Home-Anwendungen orientiert sich am „Internet Users’ Information Privacy Concern (IUIPC)-Ansatz“ von *Malhotra et al. *(2004). Der IUIPC-Ansatz nennt die individuelle Vertrauensüberzeugung („trusting beliefs“, TB) und die individuelle Risikoüberzeugung („risk beliefs“, RB) als relevante Faktoren, die die Einstellung gegenüber internetbasierten Technologien in Bezug auf eine Wahrung der Privatsphäre erklären (Malhotra et al. 2004).

Im betrachteten Zusammenhang beschreibt die individuelle Vertrauensüberzeugung die Einschätzung, inwieweit ein Unternehmen mit den übermittelten, mitunter sensiblen Daten eines Nutzers sorgsam umgeht und gegen unberechtigte Zugriffe schützt (Wu et al. 2012). Die Bedeutung von Vertrauen lässt sich damit erklären, dass Nutzer mit der Übermittlung persönlicher Daten gegenüber dem Technologieanbieter einen Vertrauensvorschuss hinsichtlich des Datenumgangs leisten. So fehlen aufgrund der Neuartigkeit des Technologieangebots vielfach Möglichkeiten, mit Hilfe eigener Erfahrungen die Vertrauenswürdigkeit des Anbieters in Bezug auf den tatsächlichen Datenumgang zu bewerten (Zhou 2014). Individuelles Vertrauen kann in dieser Konstellation an die Stelle eigener Erfahrungen treten und einen Beitrag dazu leisten, die durch fehlende Erfahrungen entstehende Unsicherheitssituation bei der Nutzung internetbasierter Technologien abzuschwächen (Hernández-Ortega 2011). Hypothese H3a unterstellt daher folgenden Wirkungszusammenhang:

*H3a: Je höher das individuelle Vertrauen in den Anbieter digitaler Finanzassistenten ist, desto positiver ist die Einstellung zur Nutzung digitaler Finanzassistenten.*

Die individuelle Risikoüberzeugung als weiteres wichtiges Element des IUIPC-Ansatzes beschreibt die Erwartung, welche negativen Konsequenzen mit der Nutzung internetbasierter Technologien verbunden werden (Malhotra et al. 2004; Slade et al. 2015). Erwartete Risiken, die im Zusammenhang mit der Übermittlung persönlicher Daten über das Internet auftreten, können als eine Kostenkategorie interpretiert werden, die im Rahmen einer Kosten-Nutzen-Abwägung die Entscheidung beeinflussen, eine angebotene Technologie zu nutzen oder abzulehnen (Juric et al. 2015). Je stärker dabei die individuelle Risikoeinschätzung ausgeprägt ist, desto ungünstiger gestaltet sich der Kosten-Nutzen-Vergleich hinschlich der Entscheidung, eine Technologie zu nutzen. Daraus folgt nachstehende Hypothese:

*H3b: Je höher die individuelle Risikoeinschätzung bezüglich der Nutzung digitaler Finanzassistenten ist, desto negativer ist die Einstellung zur Nutzung digitaler Finanzassistenten.*

**Figure 2:**
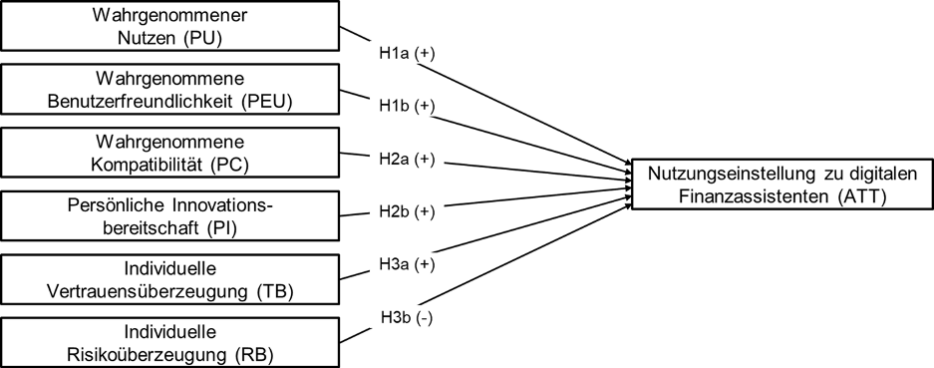

Die folgende Übersicht fasst das Untersuchungsmodell zusammen, mit dem die Einstellung zur Nutzung von Financial-Home-Anwendungen untersucht wird (Abb. 2).

## Empirische Untersuchung der Nutzungseinstellung zu digitalen Finanzassistenten

### Untersuchungsdesign

Für die vorliegende Untersuchung wurden Personen befragt, die sich in ihrem privaten Haushalt bezüglich Versicherungsangelegenheiten als Allein- oder zumindest Mit-Entscheider bezeichnen. Mit dieser Vorselektion soll sichergestellt werden, dass die Untersuchungsteilnehmer über hinreichende Kenntnisse verfügen, um Fragen zur Organisation der persönlichen Finanz- und Versicherungssituation mittels vergleichsweise neuartiger Financial-Home-Anwendung beantworten zu können. Für die Datenerhebung wurde ein Marktforschungsinstitut beauftragt, das die Befragung im Zeitraum Juni bis Juli 2021 auf Basis eines Online-Fragebogens durchführte. Nach einer Plausibilisierung und Bereinigung (etwa hinsichtlich sogenannter Straight-Liner oder Speeder; z. B. Prill 2017) umfasst die Stichprobe für die Modellüberprüfung 2054 vollständige Datensätze, die folgende soziodemografische Struktur und Erfahrungen mit digitalen Finanzassistenten aufweist (Tab. 1).

**Table 1:** 

Stichprobengröße: *n* = 2054		Prozent
Geschlecht	Männlich	48,7
	Weiblich	51,3
	Divers	0,0
Alter	Bis 25 Jahre	3,1
	26 bis 35 Jahre	15,3
	36 bis 45 Jahre	16,4
	46 bis 55 Jahre	25,8
	56 bis 65 Jahre	29,3
	66 und älter	10,1
Rolle bzgl. Versicherungsangelegenheiten	Allein-Entscheider	65,5
	Mit-Entscheider	34,5
Nutzung digitaler Finanzassistent^a^	Nutze ich häufiger	0,3
	Habe ich schon einmal genutzt	1,8
	Habe ich schon einmal von gehört	6,2
	Kenne ich nicht und nutze ich nicht	91,7

^a^ Abgefragt über Nutzung real existierender Financial-Home-Anwendungen, Mehrfachnennung möglich

Im Fragebogen werden die latenten Einstellungskonstrukte aus dem theoretisch abgeleiteten Modell (Kap. 2) mit Multi-Item-Messansätzen operationalisiert. Um eine hohe Validität sicherzustellen, orientieren sich die Fragen-Items an vorherigen Forschungsarbeiten, erhalten aber teilweise eine Erweiterung um plausibel erscheinende Items. Dabei werden die Formulierungen an den konkreten Untersuchungsgegenstand angepasst. Die Messung aller Frage-Items erfolgt jeweils mit einer 5‑Punkt-Likert-Skala mit Ausprägungen von 1 („stimme voll und ganz zu“) bis 5 („stimme überhaupt nicht zu“).^4^

### Auswertungsmethode und Untersuchungsergebnisse

Mit dem vorliegenden Modell soll festgestellt werden, ob und ggf. in welcher Stärke die verschiedenen theoretisch hergeleiteten Konstrukte Einfluss auf die Nutzungseinstellung zu digitalen Finanzassistenten nehmen. Zur Analyse des Kausalzusammenhangs mehrerer unabhängiger Variablen auf eine abhängige Variable wird die Methode der multiplen linearen Regression verwendet. Multiple Regressionsanalysen ermitteln nicht nur die Wirkung einer jeden unabhängigen auf die abhängige Variable separat. Über die Berücksichtigung von Interkorrelationen wird zusätzlich der relative Einfluss der betrachteten unabhängigen Variablen untereinander erklärt (z. B. Allison 1998; Rasch et al. 2014). Alle Berechnungen wurden mit SPSS 27.0 durchgeführt.

Bevor mittels multipler Regressionsanalysen das betrachtete Modell auf Kausalität untersucht werden kann, ist zunächst sicherzustellen, dass die Fragen-Items die jeweils betrachteten latenten Einstellungskonstrukte zuverlässig (Reliabilität) und inhaltlich zutreffend (Validität) messen. Für eine solche Gütebeurteilung der verwendeten Messinstrumente wird auf Konstruktebene die Interne-Konsistenz-Reliabilität und auf Indikatorenebene die Konvergenzvalidität herangezogen (Murphy 2009; Bieling 2011).

Als Maß für die interne Konsistenz eines latenten Einstellungskonstrukte wird deren Cronbachs Alpha sowie die Item-Trennschärfe, d. h. die korrigierte Item-to-Total-Korrelation der einem Konstrukt zugehörigen Items verwendet. Um die Reliabilitäts-Grenzwerte für Cronbachs Alpha (0,7; z. B. Nunnally und Bernstein 1994) und für die Item-Trennschärfe (0,4; z. B. Moosbrugger und Kelava 2020) zu erfüllen, werden im Rahmen eines iterativen Prozesses bei zwei Konstrukten Items aus dem Modell eliminiert (PC4, PC5, PC6, PC7, TB7, TB8)^5^. Um im nächsten Schritt die Konvergenzvalidität zu beurteilen, werden die so bereinigten latenten Einstellungskonstrukte separat einer explorativen Faktorenanalyse unterzogen. Damit wird sichergestellt, dass die einzelnen Items als Erklärungsindikatoren eines Konstrukts nur auf dieses alleine laden und damit Eindimensionalität aufweisen (Homburg und Giering 1996). Sofern diese Forderung erfüllt ist, sollte im Sinne der Konvergenzvariabilität die durch diesen Faktor erklärte Varianz aller ihm zugeordneten Indikatorvariablen mindestens 50 % betragen (Homburg und Giering 1996). Bezüglich der Konvergenzvalidität ist außerdem sicherzustellen, dass alle einem Faktor zugeordneten Indikatoren eine ausreichende Faktorladung aufweisen (mindestens 0,4; z. B. Gerbing und Anderson 1988). Tab. 2 zeigt, dass die verwendeten Messinstrumente die geforderten Gütekriterien hinsichtlich Reliabilität und Validität erfüllen.

**Table 2:** 

Einstellungskonstrukt	Item	Kommunalität	Item-Trennschärfe	Cronbachs Alpha	Faktorladung	Erklärte Varianz (%)
Grenzwert Gütekriterium			(≥ 0,4)	(≥ 0,7)	(≥ 0,4)	(≥ 50)
*Nutzungseinstellung (ATT)*	ATT1	0,89	0,89	*0,944*	0,94	*85,6*
	ATT2	0,89	0,90		0,94	
	ATT3	0,79	0,81		0,89	
	ATT4	0,86	0,87		0,93	
*Wahrgenommener Nutzen (PU)*	PU1	0,82	0,86	*0,947*	0,90	*79,0*
	PU2	0,82	0,86		0,90	
	PU3	0,71	0,77		0,84	
	PU4	0,76	0,81		0,87	
	PU5	0,79	0,84		0,89	
	PU6	0,85	0,88		0,92	
*Wahrgenommene Benutzerfreundlichkeit (PEU)*	PEU1	0,77	0,74	*0,904*	0,88	*84,0*
	PEU2	0,88	0,84		0,94	
	PEU3	0,88	0,85		0,94	
*Wahrgenommene* *Kompatibilität (PC)*	PC1	0,85	0,83	*0,898*	0,92	*83,3*
	PC2	0,85	0,82		0,92	
	PC3	0,79	0,76		0,89	
*Individuelle Innovations-bereitschaft (PI)*	PI1	0,83	0,81	*0,872*	0,91	*73,0*
	PI2	0,77	0,76		0,88	
	PI3	0,49	0,54		0,70	
	PI4	0,83	0,81		0,91	
*Individuelle Vertrauens-überzeugung (TB)*	TB1	0,84	0,88	*0,948*	0,92	*79,4*
	TB2	0,77	0,82		0,88	
	TB3	0,77	0,82		0,87	
	TB4	0,81	0,85		0,90	
	TB5	0,81	0,85		0,90	
	TB6	0,77	0,82		0,88	
*Individuelle Risiko-überzeugung (RB)*	RB1	0,81	0,77	*0,813*	0,90	*66,2*
	RB2	0,77	0,72		0,88	
	RB3	0,74	0,69		0,86	
	RB4	0,32	0,40		0,57	

Für die Quantifizierung des gemessenen Einstellungskonstrukts im Gesamtmodell werden aus den Frage-Items jeweils gewichtete Summenindizes gebildet. Um die relative Bedeutung eines jeden Items für das jeweilige Konstrukt abzubilden, dient dessen Kommunalität im betrachteten Konstrukt als Gewichtungsfaktor. Für das so berechnete Gesamtmodell kann mit einem R^2^ = 0,76 (korrigiertes R^2^ = 0,76) eine hohe Anpassungsgüte festgestellt werden (Cohen 1988). Die betrachteten unabhängigen Variablen erklären damit zusammen 76 % der Varianz der abhängigen Variablen „Nutzungseinstellung zu digitalen Finanzassistenten“. Sie weisen zudem einen signifikanten Einfluss auf (F(6, 96) = 527,67, *p* < 0,01). Daneben kann eine Autokorrelation auf Basis der Durbin-Watson-Statistik (= 2,091) ausgeschlossen werden (Eckey et al. 2011). Hinweise auf unerwünschte Effekte durch Multikollinearität zwischen den unabhängigen Variablen sind ebenfalls nicht gegebenen (jeweils VIF-Werte < 10; Wooldridge 2008).

**Figure 3:**
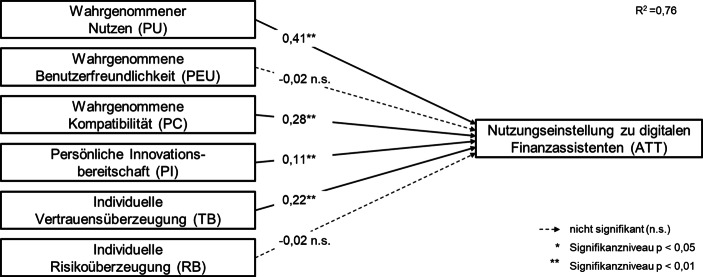

Auch das Gesamtmodell zeigt damit eine hohe Eignung hinsichtlich der angelegten Gütekriterien. Entsprechend kann mit dem Modell und seinen verwendeten Messinstrumenten die Hypothesenüberprüfung und Ergebnisinterpretation erfolgen. Eine Übersicht über die Ergebnisse der multiplen linearen Regression gibt Abb. 3.

Es ist zu erkennen, dass mit H1a, H2a, H2b und H3a vier der insgesamt sechs theoretisch abgeleiteten Hypothesen bestätigt werden. So haben der wahrgenommene Nutzen (PU), die wahrgenommene Kompatibilität (PC), die individuelle Innovationsbereitschaft (PI) sowie die individuelle Vertrauensüberzeugung einen signifikanten Einfluss auf die Nutzungseinstellung zu digitalen Finanzassistenten (ATT) und wirken auf diese jeweils positiv. Dabei geht von H1a (β = 0,41, *p* < 0,01) der vergleichsweise stärkste positive Einfluss aus, während H2a (β = 0,28, *p* < 0,01) und H3a (β = 0,22, *p* < 0,01) moderat positiv wirken und bei H2b (β = 0,11, *p* < 0,01) lediglich ein schwacher positiver Zusammenhang nachweisbar ist.

Die Ergebnisse der Hypothesenüberprüfung stimmen insgesamt mit den Befunden aus Untersuchungen zur Akzeptanz digitaler, onlinebasierter Finanzdienstleistungsangebote überein. Vor allem jüngere Studien heben ebenfalls den wahrgenommenen Nutzen des Angebots und das Vertrauen in den Anbieter als relevante Einflussgrößen für die Nutzungseinstellung hervor (Gebert-Persson et al. 2019; Rodríguez Cardona et al. 2021). Zudem zeigen andere Studien, dass neben der Nutzenerwartung auch die Kompatibilität mit dem persönlichen Lebensstil sowie die individuelle Innovationsbereitschaft positiv auf die Nutzungseinstellung gegenüber dem Angebot digitaler Finanzdienstleistungsservices wirken (z. B. Yang et al. 2012; Pham und Ho 2015; Ginner 2018).

Dagegen konnte in der vorliegenden Untersuchung für die wahrgenommene Benutzerfreundlichkeit (PEU) sowie für die individuelle Risikoüberzeugung (RB) weder ein signifikanter Einfluss noch ein relevanter Zusammenhang bezüglich der Nutzungseinstellung gegenüber digitalen Finanzassistenten festgestellt werden. Die Hypothesen H1b (β = −0,02, *p* = 0,52) und H3b (β = −0,02, *p* = 0,36) werden daher verworfen. Vor allem der fehlende Einfluss der wahrgenommenen Benutzerfreundlichkeit ist insofern bemerkenswert, als dieses Konstrukt im ursprünglichen TAM eine zentrale Rolle für die Nutzungseinstellung spielt (Abschn. 2.2).

Als eine wesentliche Begründung für dieses Ergebnis können fehlende Erfahrungen der Befragungsteilnehmer bezüglich der Nutzung digitaler Finanzassistenten angenommen werden (Tab. 1). Während andere der abgefragten Konstrukte über Aspekte erfasst werden, bei denen sich Erfahrungsdefizite zumindest teilweise durch eine hohe Generalisierbarkeit kompensieren lassen (z. B. Zeitersparnis bei der Bewertung des wahrgenommenen Nutzens), hängt die wahrgenommene Benutzerfreundlichkeit deutlich stärker von der konkreten Ausgestaltung der betrachteten Anwendung ab (z. B. konkrete Ausgestaltung von Benutzeroberflächen oder Menüführung). Die Vernachlässigung der Benutzerfreundlichkeit in Bezug auf die Nutzungseinstellung stellt dann weniger das Ergebnis einer inhaltlichen Kosten-Nutzen-Bewertung dar, sondern resultiert schlichtweg aus mangelnden Erfahrungen in der tatsächlichen Bedienung digitaler Finanzassistenten (ähnlich in einem anderen Kontext Stoel und Hye Lee 2003). Daneben lässt sich die geringe Bedeutung der Benutzerfreundlichkeit auch mit der Verbreitung mobiler Endgeräte und mobiler Anwendungen erklären, bei deren Nutzung die Benutzerfreundlichkeit zunehmend als Basisanforderung wahrgenommen wird, die lediglich dazu beiträgt, eine Ablehnung der betrachteten technischen Anwendung zu verhindern, selbst aber keinen Mehrwert im Rahmen der Kosten-Nutzen-Bewertung stiftet (Li und You 2016). Entsprechend nimmt die Bedeutung der Benutzerfreundlichkeit der eigentlichen Anwendung im Rahmen der Nutzungseinstellung ab.

Hinsichtlich der individuellen Risikoüberzeugung kann der fehlende signifikante Einfluss ebenfalls mit der geringen tatsächlichen Nutzungserfahrung digitaler Finanzassistenten begründet werden. So gestaltet sich die Risikoeinschätzung einer Technologie als schwierig, wenn aufgrund fehlender Erfahrungswerte nicht nachvollzogen werden kann, wie und in welcher Form Daten konkret erhoben und online übermittelt werden. Daneben können die Ergebnisse bezüglich der individuellen Risikoüberzeugung auch als Ergebnis eines Gewöhnungseffekts bei der Nutzung internetbasierter Technologien interpretiert werden (Gebert-Persson et al. 2019). So lässt sich feststellen, dass in dem Maße, in dem für unterschiedliche Lebensbereiche der regelmäßige Gebrauch von Online-Anwendungen zur Selbstverständlichkeit wird (z. B. für Freizeit, Mobilität, Konsum, Finanzen), Risiken eines Online-Datentransfers zunehmend als beherrschbar oder vernachlässigbar wahrgenommen werden. Eine Verstärkung dieses Effekts lässt sich infolge der Maßnahmen zur Bekämpfung der im Jahr 2020 ausgebrochenen Covid-19-Pandemie beobachten. So zeigen erste Untersuchungen, dass als ein Ergebnis der in vielen Ländern zur Pandemiebekämpfung erlassenen Kontaktbeschränkungen eine steigende Aufgeschlossenheit gegenüber digitalen, onlinebasierten Finanzdienstleistungsangeboten feststellbar ist (z. B. Sreelakshmi und Prathap 2020; Zhao und Bacao 2021).

### Explorative Modellmodifikation

Als Ergebnis der Gütebeurteilung der verwendeten Messinstrumente bleiben beim theoretisch abgeleiteten Ursprungsmodell insgesamt sechs erhobene Items unberücksichtigt (Abschn. 3.2). Um zu überprüfen, ob diese eliminierten Frage-Items möglicherweise im Zusammenhang mit anderen bestehenden oder neu zu bildenden Einstellungskonstrukten einen zusätzlichen Erklärungsbeitrag für die Nutzungseinstellung gegenüber digitalen Finanzassistenten liefern, wird auf alle in der Untersuchung erhobenen Items eine explorative Faktorenanalyse angewendet.

Mit einem Kaiser-Meyer-Olkin-Koeffizienten (KMO) von 0,93 (mindestens 0,6; z. B. Hair et al. 2006) und einem signifikanten Bartlett-Test (*p* < 0,01) lässt sich feststellen, dass sich der verwendete Datensatz grundsätzlich für die Durchführung einer explorativen Faktorenanalyse eignet. Für die Faktorreduktion wird in der vorliegenden Untersuchung die Hauptkomponentenanalyse angewendet, die das Ziel verfolgt, mit möglichst wenigen, voneinander unabhängige Hauptkomponenten möglichst viel Varianz in den Variablen zu erklären. Zur Trennung der Faktoren kommt die Varimax-Rotation zur Anwendung (z. B. Backhaus et al. 2016).

Insgesamt lassen sich sechs Faktoren identifizieren, von denen fünf die geforderten Gütekriterien als valide und reliable Messinstrumente erfüllen.^6^ Diese fünf Faktoren bilden die latenten Konstrukte, die als unabhängige Variablen hinsichtlich der Erklärung der Nutzungseinstellung zu digitalen Finanzassistenten (ATT) als abhängige Variable untersucht werden. Neben Items, deren Zusammenfassung als Faktoren eine geringe Validität und Reliabilität aufweisen (PC6, PC7), wurden weitere vier Items eliminiert (PEU1, PEU2, PEU3, PC2), die eine heterogene oder schwache Faktorladung aufweisen (z. B. Hair et al. 2006). Der explorativ induzierte Ausschluss der Items PEU1, PEU2, PEU3 bestätigt damit insofern das Ergebnis des ursprünglichen Modells, in dem für die wahrgenommene Benutzerfreundlichkeit (PEU) keine signifikante Bedeutung bezüglich der Nutzungseinstellung gegenüber digitalen Finanzassistenten festgestellt werden konnte (Abschn. 3.2).

**Figure 4:**
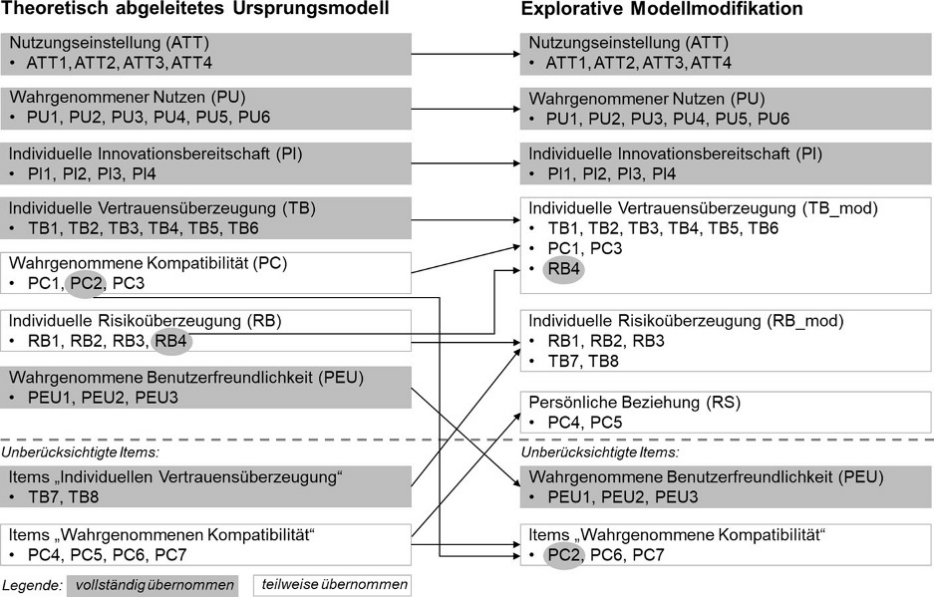

Die auf Basis der explorativen Faktorenanalyse vorgenommene Modellmodifikation hinsichtlich der Item-Konstrukt-Zuordnung zeigt Abb. 4.

Da auch die Modellmodifikation den Einfluss mehrerer Variablen auf eine abhängige Variable überprüft, wird für die Kausalanalyse ebenfalls eine multiple lineare Regression angewendet. Für die Quantifizierung der Konstrukte im Gesamtmodell werden wiederum mit den Item-Kommunalitäten gewichtete Summenindizes gebildet. Auch das explorativ modifizierte Modell zeigt bezüglich der unabhängigen Variablen eine hohe Anpassungsgüte (R^2^ und korrigiertes R^2^ = 0,75). Auf die Nutzungseinstellung zu digitalen Finanzassistenten als abhängige Variable ist ein signifikanter Einfluss feststellbar (F(5,96) = 630,743, *p* < 0,01). Autokorrelation und Multikollinearität können auf Basis der Durbin-Watson-Statistik (= 2,026) und der VIF-Werte (jeweils < 10) ausgeschlossen werden.

**Figure 5:**
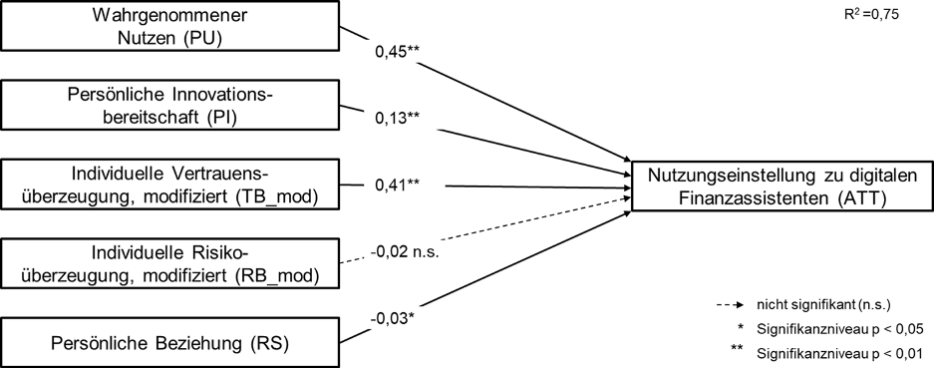

Die durchgeführte multiple Regressionsanalyse bestätigt hinsichtlich des wahrgenommenen Nutzens (PU), der persönlichen Innovationsbereitschaft (PI) und der individuellen Risikoüberzeugung (RB) die Ergebnisse des ursprünglichen Modells (Abb. 5). Auch in der Modellmodifikation geht unter den betrachten Einflussfaktoren vom wahrgenommenen Nutzen (PU) der vergleichsweise stärkste signifikante Einfluss aus (β = 0,45, *p* < 0,01). Die persönliche Innovationsbereitschaft (PI) zeigt zwar einen signifikanten Einfluss, der aber wie im ursprünglichen Modell nur schwach positiv ausgeprägt ist (β = 0,13, *p* < 0,01). Diese beiden Konstrukte wurden mit denselben Frage-Items gemessen wie im Ursprungsmodell. Hingegen wird die Messung der individuellen Risikobereitschaft vor allem durch die Aufnahme von zwei Items modifiziert, die im theoretisch abgeleiteten Modell keine Berücksichtigung fanden (TB7, TB8). Trotz dieser explorativen Modifikation bestätigt sich, dass zwischen modifizierter individueller Risikobereitschaft (RB_mod) und der Nutzungseinstellung gegenüber digitalen Finanzassistenten weder ein signifikanter noch ein starker Zusammenhang feststellbar ist (β = −0,02, *p* = 0,24).

Unterschiede zwischen dem ursprünglichen Theoriemodell und seiner explorativen Modifikation lassen sich hingegen bezüglich des Konstrukts der individuellen Vertrauenseinstellung (TB) feststellen. So extrahiert die Faktorenanalyse ein Einstellungskonstrukt, das die Messung der individuellen Vertrauensüberzeugung (TB1, TB2, TB3, TB4, TB5, TB6) mit Items der ursprünglichen Konstrukte der wahrgenommenen Kompatibilität (PC1, PC3) und der individuellen Risikoüberzeugung (RB4) verbindet. Die so modifizierte individuelle Vertrauensüberzeugung (TB_mod) liefert die zweitstärkste Erklärung für eine positive Nutzungseinstellung gegenüber digitalen Finanzassistenten (β = 0,41, *p* < 0,01). Vor allem die Erweiterung um Items zur Messung der wahrgenommenen Kompatibilität deutet darauf hin, dass zwischen Lebenseinstellung und -stil eines Nutzers und dessen Vertrauen in einen Technologieanbieter ein Zusammenhang besteht. So erscheint es durchaus plausibel, dass Anwender, die eine generell positive Einstellung gegenüber digitalen Angeboten aufweisen, auch den Anbietern neuer Technologien in Bezug auf die Vertrauenswürdigkeit der angebotenen Leistung grundsätzlich aufgeschlossen gegenüberstehen (Kaabachi et al. 2019).

Als weiterer Erkenntnisgewinn liefert die explorative Modellmodifikation Hinweise darauf, dass die Nutzungseinstellung gegenüber digitalen Finanzassistenten in dem Maße sinkt, in dem eine direkte persönliche Beziehung („personal relationship“, RS) bei der Abwicklung von Finanzangelegenheiten als relevant wahrgenommen wird. Dieses im Vergleich zum theoretisch abgeleiteten Modell zusätzliche Konstrukt umfasst zwei Items (PC4, PC5), die im Ursprungsmodell nicht berücksichtigt wurden (Abschn. 3.2). Auch wenn sich mit dem verwendeten, explorativ erhobenen Messinstrument lediglich ein schwach negativer Zusammenhang feststellen lässt (β = −0,03, *p* < 0,05), deckt sich diese Erkenntnis mit den Ergebnissen von Studien, die die Bedeutung persönlicher Beziehungen im Vertrieb und in der Betreuung vor allem beratungsintensiver Finanzprodukte belegen (z. B. für die Altersvorsorge Bode und Wilke 2014).

## Diskussion der Untersuchungsergebnisse und Ausblick

### Handlungsempfehlungen für die Versicherungspraxis

In der vorliegenden Untersuchung wurde zunächst ein theoretisches Modell zur Untersuchung der Nutzungseinstellung gegenüber digitalen Finanzassistenten entwickelt und empirisch überprüft. Die anschließende explorative Faktorenanalyse sollte dazu beitragen, zusätzliche, im Rahmen der theoretischen Modellbildung möglicherweise unberücksichtigte Zusammenhänge in den betrachteten Einstellungskonstrukten aufzudecken. Die Konstrukte entstammen theoretischen Modellen zur Technologieakzeptanzforschung (TAM und seine Erweiterungen) und zur Untersuchung von Privatsphärenbedenken bei der Nutzung internetbasierter Technologien (IUIPC-Modell). Erkenntnisse dieser Ansätze bilden die Grundlagen für die Hypothesengenerierung in Bezug auf die Nutzungseinstellung gegenüber digitalen Finanzassistenten. Für die Hypothesenüberprüfung des theoretisch abgeleiteten als auch für die Kausalanalyse des explorativ modifizierten Modells kam jeweils eine multiple lineare Regression zur Anwendung. Die Ergebnisse der Regressionsanalysen können der Unternehmenspraxis als folgende Handlungsempfehlungen dienen:

Der wahrgenommene Nutzen zeigt sich als wichtiger, signifikanter Einflussfaktor für die Absicht eines Anwenders, mit einem digitalen Finanzassistenten seine Finanzangelegenheiten gesamthaft über eine integrierte Online-Anwendung abzuwickeln. Dieses Ergebnis ist insofern nachvollziehbar, als das Angebot einer solchen Online-Anwendung kundenseitig überhaupt erst dann wahrgenommen werden dürfte, wenn aus ihrer Verwendung ein erwarteter Nutzen resultiert. Der wahrgenommene Nutzen kann von der Unternehmenspraxis damit als Basisanforderung bezüglich der inhaltlichen Gestaltung digitaler Finanzassistenten interpretiert werden, deren Erfüllung aus Nutzersicht die Grundvoraussetzung für die Inanspruchnahme bildet.

Damit das Angebot digitaler Finanzassistenten auch tatsächlich angenommen wird, muss die Anwendung inhaltlich folglich so gestaltet sein, dass potenzielle Nutzer in ihrer Verwendung einen persönlichen Nutzen sehen oder diesen höher bewerten als eine im Vergleich analoge oder über mehrere Online-Anwendungen verschiedener Banken und Versicherer verteilte Darstellung der persönlichen Finanzen und Versicherungen. Solche Nutzenaspekte können sich etwa in einer transparenten Übersicht der gesamten Finanz- und Absicherungssituation oder in einer zeit- und aufwandsarmen Erledigung aller Finanzangelegenheiten über eine Anwendung dokumentieren. Anbieter digitaler Finanzassistenten können die Präsentation entsprechender Vorteile ihres Serviceangebots über entsprechende Kommunikations- und Marketingmaßnahmen unterstützen. Ein weiterer wahrgenommener Nutzen könnte sich auf finanzielle Vorteile bei der Verwendung digitaler Finanzassistenten beziehen. Solche Vorteile sind denkbar, wenn die Financial-Home-Anwendung etwa über Funktionen verfügt, hinterlegte Finanzdaten für das Aufzeigen von Sparpotenzialen (z. B. Hinweise auf preiswertere Versicherungsprodukte bei vergleichbarem Leistungsumfang) oder das Identifizieren möglicher Deckungslücken (z. B. Absicherung der Arbeitskraft beim Fehlen entsprechender Produktlösungen) einzusetzen.

Als weiterer signifikanter Treiber der Einstellung zu digitalen Finanzassistenten wurde die Vertrauensüberzeugung nachgewiesen. Damit Financial-Home-Anwendungen tatsächlich genutzt werden, erscheint es für die Anbieterseite daher vorteilhaft, gegenüber potenziellen Nutzern eine Vertrauensposition aufzubauen. Vertrauensbildend kann etwa eine Zertifizierung der Anwendung hinsichtlich Datenschutz und -sicherheit (z. B. DSGVO-Konformität) durch eine dem Nutzer bekannte externe Institution (z. B. TÜV) wirken (Juric et al. 2015). Vor allem Banken und Versicherer können außerdem ihre Rolle als etablierte Marktteilnehmer dazu verwerten, sich beim Angebot digitaler Finanzassistenten als vertrauensvolle Akteure zu positionieren. So verfügen solche Unternehmen gegenüber jüngeren Marktteilnehmern, wie es beispielweise Fin- oder Insurtechs zumeist sind, i. d. R. über eine vertrauensunterstützende Reputation auf Basis langfristiger Kundenbeziehungen (Bömer und Maxin 2018).

Neben diesen grundsätzlichen Empfehlungen ist die Bedeutung des wahrgenommenen Nutzens und des Anbietervertrauens zudem vor dem Hintergrund der ebenfalls signifikanten Einflussfaktoren der persönlichen Innovationsbereitschaft und der wahrgenommenen Kompatibilität zu betrachten. So deuten die Untersuchungsergebnisse darauf hin, dass die Nutzungseinstellung zu digitalen Finanzassistenten auch von Treibern abhängt, die in der individuellen Persönlichkeit des jeweiligen Anwenders liegt. Dabei lassen die Ergebnisse den Schluss zu, dass sich solche Nutzer grundsätzlich leichter zur Verwendung digitaler Finanzassistenten bewegen lassen, deren Neugier am Ausprobieren neuer Technologien stark ausgeprägt ist und ein solches Verhalten als passend zu ihrem individuellen Lebensstil wahrnehmen. Sind diese Persönlichkeitsmerkmale hingegen weniger stark ausgeprägt, können stattdessen der anbieterseitige Aufbau einer Vertrauensposition und das Aufzeigen von Nutzenaspekten dazu beitragen, potenzielle Nutzer von digitalen Finanzassistenten zu überzeugen. Aus dieser Interpretation der Studienergebnisse lässt sich für die Unternehmenspraxis eine differenzierte Ansprache potenzieller Kunden bezüglich der Verwendung eines angebotenen digitalen Finanzassistenten ableiten: Während gegenüber innovationsfreudigen, neuen Technologien aufgeschlossenen Anwendern die Neuartigkeit digitaler Finanzassistenten in den Vordergrund gestellt werden kann, scheint bei Nutzern, deren Einstellung in dieser Hinsicht weniger stark ausgeprägt ist, eine anbietervertrauensschaffende Präsentation der wesentlichen Nutzenaspekte eines entsprechendes Online-Angebots zielführender zu sein.

Abschließend sei darauf hingewiesen, dass der in dieser Studie fehlende Einfluss der wahrgenommenen Benutzerfreundlichkeit nicht zum Schluss führen sollte, dass eine intuitive und benutzerfreundliche Gestaltung von Financial-Home-Anwendungen für die Praxis keine Bedeutung spielt. Vielmehr ist anzunehmen, dass die entsprechenden Ergebnisse zumindest in Teilen mit fehlenden Erfahrungen in der tatsächlichen Nutzung digitaler Finanzassistenten zu erklären sind. So legen Untersuchungen zu digitalen Finanzdienstleistungsangebote, die aufgrund ihrer Verbreitung das Sammeln für eine Beurteilung hinreichender Nutzungserfahrungen erlauben (z. B. Self-Service-Angebote auf Versicherungsportalen, Online-Banking), einen entsprechenden positiven Zusammenhang zwischen wahrgenommener Benutzerfreundlichkeit und Nutzungsakzeptanz nahe (Juric et al. 2015, Vuković et al. 2019).

### Studienlimitationen

Die Ergebnisse der vorliegenden Untersuchung und daraus abgeleitete Handlungsempfehlungen für die Unternehmenspraxis sind vor dem Hintergrund folgender Einschränkungen zu betrachten:

Bei den untersuchten Einstellungskonstrukten handelt es sich um theoretisch abgeleitete Einflussfaktoren, deren Bedeutung allgemein für die Akzeptanz neuer Technologien im Finanzdienstleistungsbereich in verschiedenen Studien bestätigt wurde. Es kann allerdings nicht ausgeschlossen werden, dass weitere Konstrukte existieren, die im Besonderen für die Nutzung digitaler Finanzassistenten einen zusätzlichen Erklärungsbeitrag liefern.

Über die Messung verschiedener Einstellungskonstrukte untersucht die vorliegende Studie die Absicht, einen digitalen Finanzassistenten zu nutzen, nicht aber die Realisierung dieser Nutzungsabsicht. Zwar legen verschiedene Untersuchungen nahe, dass die Nutzungsabsicht einen Prädiktor für das Nutzungsverhalten darstellt (Abschn. 3.2), eine abschließende Aussage über die tatsächliche Verwendung digitaler Finanzassistenten bei Erfüllung der als signifikant identifizierten Einflussfaktoren kann auf Basis der Untersuchungsergebnisse aber nicht getroffen werden.

Die Messung der tatsächlichen Verwendung digitaler Finanzassistenten gestaltet sich mit der vorliegenden Studie auch insofern als schwierig, als infolge ihrer Neuartigkeit zum Zeitpunkt der Datenerhebung von einer geringen Nutzerdurchdringung ausgegangen werden muss. Das damit verbundene Defizit an tatsächlichen Nutzungserfahrungen kann möglicherweise zu Verzerrungen bei der Item-Bewertung im Sinne eines „Response Bias“ führen (z. B. Furnham 1986).

Mit der Berücksichtigung verschiedener Einstellungskonstrukte analysiert die vorliegende Untersuchung schließlich den direkten Einfluss mehrerer unabhängiger Variablen auf eine abhängige Variable. Verkettete Ursache-Wirkungs-Zusammenhänge der Einstellungskonstrukte, die in Bezug auf die Nutzungseinstellung einen indirekten Einfluss entfalten (z. B. Pfadkorrelationen), werden somit von der Untersuchung ausgeschlossen.

### Ausblick auf künftige Forschungsansätze

Die aufgezeigten Limitationen dieser Untersuchungen bieten gleichzeitig Ansatzpunkte für künftige Forschungen. So könnten weitere Studien untersuchen, ob noch zusätzliche Konstrukte den Erklärungsgehalt des theoretisch abgeleiteten und des explorativ modifizierten Modells erhöhen. Erste Hinweise hierauf liefert die durchgeführte Faktorenanalyse z. B. hinsichtlich der Bedeutung persönlicher Beziehungen. Diese Erkenntnisse könnten über eine theoriegeleitete Erweiterung des entsprechenden Item-Sets in Folgeuntersuchungen überprüft und vertieft werden können.

Mit einer zunehmenden Verbreitung digitaler Finanzassistenten könnten solche Studien auch das tatsächliche Nutzungsverhalten untersuchen. Eine solche Erweiterung könnte zu einer Validierung der Ergebnisse der vorliegenden Untersuchung beitragen, die zwar einen Zusammenhang zwischen der Absicht und der Verwendung digitaler Finanzassistenten unterstellt, die tatsächliche Nutzungsentscheidung aber nicht erfasst.

Schließlich deuten die Ergebnisse dieser Studie darauf hin, dass die Akzeptanz digitaler Finanzassistenten von individuellen Persönlichkeitsmerkmalen abhängt, aus denen sich Hinweise auf bestimmte Zielgruppen ableiten lassen. Um die für Financial-Home-Anwendungen relevanten Zielgruppen weiter zu schärfen, könnten Folgeuntersuchungen zusätzliche nutzerbezogene Informationen berücksichtigen. Hierzu zählen z. B. sozidemografische Daten, Besitz von Absicherungs- und Finanzprodukten oder die Vertriebswege- und Kommunikationskanalpräferenz.

## Appendix

**Table 3:**

Konstrukt im Ursprungsmodell	Code	Item	Quelle
Nutzungseinstellung digitale Finanzassistenten (ATT)	ATT1	Ein digitaler Finanzassistent ist meiner Meinung nach sinnvoll	Eigenentwicklung
	ATT2	Ein digitaler Finanzassistent ist meiner Meinung nach positiv zu bewerten	
	ATT3	Ich beabsichtige, einen digitalen Finanzassistent in Zukunft zu verwenden	
	ATT4	Bei einem digitalen Finanzassistent sehe ich mehr Vorteile als Nachteile	
Wahrgenommener Nutzen (PU)	PU1	Durch die Nutzung eines digitalen Finanzassistenten habe ich meine gesamte Finanzsituation jederzeit im Blick (z. B. Bankenkonten, Versicherungsverträge)	Eigenentwicklung
	PU2	Durch die Nutzung eines digitalen Finanzassistenten bin ich in Bezug auf meine Finanzsituation immer auf dem aktuellsten Stand (z. B. Bankkonten, Versicherungsverträge)	
	PU3	Durch die Nutzung eines digitalen Finanzassistenten erhalte ich passgenaue Finanz- und Versicherungsangebote	
	PU4	Durch die Nutzung eines digitalen Finanzassistenten werden mir Sparpotenziale aufgezeigt	
	PU5	Durch die Nutzung eines digitalen Finanzassistenten spare ich sehr viel Zeit	
	PU6	Durch die Nutzung eines digitalen Finanzassistenten kann ich meine Finanzangelegenheiten einfach und mit wenig Aufwand erledigen	
Wahrgenommene Benutzerfreundlichkeit (PEU)	PEU1	Die Nutzung eines digitalen Finanzassistenten erfordert keine besonderen Fachkenntnisse	In Anlehnung an Venkatesh 2000, Juric et al. 2015
	PEU2	Die Anpassung von individuellen Einstellungen in einem digitalen Finanzassistenten fällt mir leicht (z. B. Einbindung von Bankkonten oder Versicherungsverträgen)	
	PEU3	Die Bedienung eines digitalen Finanzassistenten fällt mir insgesamt leicht	
Wahrgenommene Kompatibilität (PC)	PC1	Die Verwendung eines digitalen Finanzassistenten passt gut zu meinem Lebensstil	In Anlehnung an Schierz et al. 2010 mit eigenen Erweiterungen
	PC2	Die Verwendung eines digitalen Finanzassistenten passt gut zu der Art und Weise, wie ich bei meinen Finanzangelegenheiten den Überblick behalte	
	PC3	Ich bevorzuge die Verwendung eines digitalen Finanzassistenten anstelle anderer Bank- oder Versicherungsportale (wie z. B. Online-Banking-App meiner Bank, Kunden-App oder Kundenportal meines Versicherers)	
	PC4	Ich befürchte, dass ich durch die Nutzung eines digitalen Finanzassistenten die gute Beziehung zu meiner Bank riskiere	
	PC5	Ich befürchte, dass ich durch die Nutzung eines digitalen Finanzassistenten die gute Beziehung zu meiner Versicherungsgesellschaft riskiere	
	PC6	Auch wenn ich einen digitalen Finanzassistenten nutze, tätige ich Überweisungen lieber direkt über meine Bank	
	PC7	Auch wenn ich einen digitalen Finanzassistenten nutze, schließe ich Versicherungen lieber direkt bei meiner Versicherungsgesellschaft ab	
Persönliche Innovationsbereitschaft (PI)	PI1	Wenn ich von einer neuen Informationstechnologie höre, suche ich nach Möglichkeiten, damit zu experimentieren	Agarwal und Prasad 1998
	PI2	Unter meinen Freund*innen/Kolleg*innen bin ich üblicherweise der/die Erste, der/die eine neue Informationstechnologie ausprobiert	
	PI3	Generell bin ich beim Ausprobieren neuer Informationstechnologien zurückhaltend	
	PI4	Es gefällt mir, mit neuen Informationstechnologien zu experimentieren	
Individuelle Vertrauensüberzeugung (TB)	TB1	Der Anbieter eines digitalen Finanzassistenten geht vertrauenswürdig mit meinen Finanzdaten um	In Anlehnung an Malhotra et al. 2004 mit eigenen Erweiterungen
	TB2	Ich gehe davon aus, dass der Anbieter eines digitalen Finanzassistenten meine Interessen beim Umgang mit meinen Finanzdaten berücksichtigt	
	TB3	Der Anbieter eines digitalen Finanzassistenten verhält sich in Bezug auf die Nutzung der von mir gelieferten Finanzdaten vorhersehbar und widerspruchsfrei	
	TB4	Der Anbieter eines digitalen Finanzassistenten geht immer ehrlich mit seinen Kunden um, wenn es um die Nutzung der von den Kunden gelieferten Finanzdaten geht	
	TB5	Ich kann darauf vertrauen, dass Angebote, die ich im digitalen Finanzassistenten erhalte, vertrauenswürdig und qualitativ geprüft sind	
	TB6	Ich kann darauf vertrauen, dass Angebote, die ich im digitalen Finanzassistenten erhalte, mindestens so gut sind wie meine aktuellen Finanzprodukte	
	TB7	Ich befürchte, bei der Nutzung eines digitalen Finanzassistenten zu viel allgemeine Werbung zu erhalten	
	TB8	Ich befürchte, bei der Nutzung eines digitalen Finanzassistenten zu viele Angebote für alternative Finanzprodukte zu erhalten	
Individuelle Risikoüberzeugung (RB)	RB1	Es ist riskant, Informationen zu meiner Finanzsituation (z. B. Bankkonten, Versicherungsverträge) online über einen digitalen Finanzassistenten zur Verfügung zu stellen	In Anlehnung an Malhotra et al. 2004
	RB2	Bei der Bereitstellung von Informationen zu meiner Finanzsituation (z. B. Bankkonten, Versicherungsverträge) online über einen digitalen Finanzassistenten bestehen einfach zu viele Unsicherheiten	
	RB3	Die Bereitstellung von Informationen zu meiner Finanzsituation (z. B. Bankkonten, Versicherungsverträge) online über einen digitalen Finanzassistenten kann zu unerwarteten Problemen führen	
	RB4	Ich fühle mich wohl dabei, wenn ich Informationen zu meiner Finanzsituation (z. B. Bankkonten, Versicherungsverträge) online über einen digitalen Finanzassistenten übermitteln kann

**Table 4:**

Item	Komponente (= Einstellungskonstrukt)					
	*Wahrgenommener Nutzen (PU)*	*Individuelle Innovations-bereitschaft (PI)*	*Individuelle Vertrauensüberzeugung, modifiziert (TB_mod)*	*Individuelle Risikoüberzeugung, modifiziert (RB_mod)*	*Persönliche Beziehung* *(RS)*	*Direkter Zugang* *(DA)* ^*b*^
PI1	–	0,879	–	–	–	–
PI2	–	0,853	–	–	–	–
PI3	–	0,548	–	–	–	–
PI4	–	0,869	–	–	–	–
PU1	0,830	–	–	–	–	–
PU2	0,795	–	–	–	–	–
PU3	0,681	–	–	–	–	–
PU4	0,736	–	–	–	–	–
PU5	0,764	–	–	–	–	–
PU6	0,821	–	–	–	–	–
PC1	–	–	0,556	–	–	–
PC3	–	–	0,577	–	–	–
TB1	–	–	0,856	–	–	–
TB2	–	–	0,780	–	–	–
TB3	–	–	0,795	–	–	–
TB4	–	–	0,841	–	–	–
TB5	–	–	0,791	–	–	–
TB6	–	–	0,773	–	–	–
RB4	–	–	0,682	–	–	–
PC4	–	–	–	–	0,911	–
PC5	–	–	–	–	0,915	–
TB7	–	–	–	0,689	–	–
TB8	–	–	–	0,604	–	–
RB1	–	–	–	0,824	–	–
RB2	–	–	–	0,796	–	–
RB3	–	–	–	0,838	–	–
PC6	–	–	–	–	–	0,802
PC7	–	–	–	–	–	0,742

Extraktionsmethode: Hauptkomponentenanalyse

Rotationsmethode: Varimax mit Kaiser-Normalisierung

^a^ Die Rotation ist in 7 Iterationen konvergiert

^b^ Faktor „Direkter Zugang“ („direct access“, DA) als Einstellungskonstrukt im Rahmen der Gütekriterien-Beurteilung verworfen

**Table 5:**

Einstellungskonstrukt	Item	Kommunalität	Item-Trenn-schärfe	Cronbachs Alpha	Faktor-ladung	Erklärte Varianz (%)
Grenzwert Gütekriterium			(≥ 0,4)	(≥ 0,7)	(≥ 0,4)	(≥ 50)
*Nutzungseinstellung (ATT)*	ATT1	0,89	0,89	*0,944*	0,94	85,6
	ATT2	0,89	0,90		0,94	
	ATT3	0,79	0,81		0,89	
	ATT4	0,86	0,87		0,93	
*Wahrgenommener Nutzen (PU)*	PU1	0,82	0,86	*0,947*	0,90	79,0
	PU2	0,82	0,86		0,90	
	PU3	0,71	0,77		0,84	
	PU4	0,76	0,81		0,87	
	PU5	0,79	0,84		0,89	
	PU6	0,85	0,88		0,92	
*Individuelle Innovations-bereitschaft (PI)*	PI1	0,83	0,81	*0,872*	0,91	73,0
	PI2	0,77	0,76		0,88	
	PI3	0,50	0,54		0,70	
	PI4	0,83	0,81		0,91	
*Individuelle Vertrauensüberzeugung, modifiziert (TB_mod)*	TP1	0,81	0,86	*0,945*	0,90	70,0
	TP2	0,72	0,79		0,84	
	TP3	0,74	0,81		0,86	
	TP4	0,79	0,84		0,89	
	TP5	0,77	0,83		0,88	
	TP6	0,75	0,82		0,86	
	PC1	0,62	0,74		0,79	
	PC3	0,55	0,68		0,74	
	RB4	0,58	0,70		0,76	
*Individuelle Risikoüberzeugung, modifiziert (RB_mod)*	RB1	0,73	0,72	*0,826*	0,85	61,1
	RB2	0,66	0,66		0,81	
	RB3	0,71	0,70		0,84	
	TP7	0,55	0,62		0,74	
	TP8	0,40	0,50		0,64	
*Persönliche Beziehung (RS)*	PC6	0,74	0,79	*0,882*	0,95	89,5
	PC7	0,74	0,79		0,95	

^a^ Extrahierter Faktor „Direkter Zugang“ („direct access“, DA) aufgrund Cronbachs Alpha < 0,7 als nicht-valides und nicht-reliables Messkonstrukt verworfen

## Footnotes

1 Die Europäische Kommission diskutiert unter dem Begriff „Open Finance“ bereits eine Ausweitung solcher Datenöffnungsregelungen auf andere Finanzdienstleister wie Versicherer (Europäische Kommission 2020). Zur Bedeutung dieser Überlegungen für deutsche Versicherer vgl. Kwasniok (2020).

2 Als Beispiele können die Anwendungen „Heymoney“ der Allianz, „fin4u“ der ALH-Gruppe oder „FinanzGuide“ der Wüstenrot & Württembergische genannt werden.

3 Ausnahmen bilden Studien vor allem von Unternehmensberatungen, die trotz ihrer interessanten Praxiseinblicke zumeist methodisch rein deskriptive Ansätze ohne Modellbezug verwenden (z. B. PricewaterhouseCoopers 2017; Oliver Wyman 2019).

4 Der Appendix gibt eine Übersicht über die verwendeten Frage-Items zur Messung der latenten Einstellungskonstrukte.

5 PC4: Ich befürchte, dass ich durch die Nutzung eines digitalen Finanzassistenten die gute Beziehung zu meiner Bank riskiere. PC5: Ich befürchte, dass ich durch die Nutzung eines digitalen Finanzassistenten die gute Beziehung zu meiner Versicherungsgesellschaft riskiere. PC6: Auch wenn ich einen digitalen Finanzassistenten nutze, tätige ich Überweisungen lieber direkt über meine Bank. PC7: Auch wenn ich einen digitalen Finanzassistenten nutze, schließe ich Versicherungen lieber direkt bei meiner Versicherungsgesellschaft ab. TB7: Ich befürchte, bei der Nutzung eines digitalen Finanzassistenten zu viel allgemeine Werbung zu erhalten. TB8: Ich befürchte, bei der Nutzung eines digitalen Finanzassistenten zu viele Angebote für alternative Finanzprodukte zu erhalten.

6 Eine Übersicht der extrahierten Faktoren sowie die Ausprägungen der einzelnen Gütekriterien der extrahierten Faktoren der Modellmodifikation findet sich im Appendix.
